# A190 THE IMPACT OF BOWEL URGENCY ON THE LIVES OF PATIENTS WITH ULCERATIVE COLITIS IN THE US AND EUROPE: COMMUNICATING NEEDS AND FEATURES OF IBD EXPERIENCES (CONFIDE) SURVEY

**DOI:** 10.1093/jcag/gwac036.190

**Published:** 2023-03-07

**Authors:** S Schreiber, A P Bleakman, M C Dubinsky, D Rubin, T Hibi, R Panaccione, T H Gibble, C Kayhan, E Flynn, C Sapin, C Atkinson, S Travis, J Jones

**Affiliations:** 1 University Hospital Schleswig-Holstein, Kiel, Germany; 2 Eli Lilly and Company, Indianapolis; 3 Mount Sinai Hospital, New York; 4 University of Chicago Medicine Inflammatory Bowel Disease Center, Chicago, United States; 5 Center for Advanced IBD Research and Treatment, Kitasato University Kitasato Institute Hospital, Tokyo, Japan; 6 University of Calgary, Calgary, Canada; 7 Eli Lilly and Company, Indianapolis, India; 8 Adelphi Real World, Bollington; 9 University of Oxford, Oxford, United Kingdom; 10 Division of Digestive Care and Endoscopy, Department of Medicine, Department of Community Health and Epidemiology, Dalhousie University, Halifax, Canada

## Abstract

**Background:**

Moderate to severe ulcerative colitis (UC) exerts a significant burden on patients’ lives. Patients with UC report that bowel urgency has a substantial negative impact on their quality of life and psychosocial functioning, however, this symptom is missing from most disease activity indices.

**Purpose:**

The Communicating Needs and Features of IBD Experiences (CONFIDE) study aims to increase understanding of the impact of symptoms, including bowel urgency, on the lives of patients (pts) with moderate to severe UC and Crohn’s disease in the United States (US), Europe (EUR), and Japan. These data focus on pts in the US and EUR.

**Method:**

Online, quantitative, cross-sectional surveys of pts with moderate to severe UC were conducted in the US and EUR (France, Germany, Italy, Spain, and UK). Data included pt perspectives on their UC symptoms and the impact on their daily lives. Moderate to severe UC was defined based on treatment, steroid use, and/or hospitalization history. Descriptive statistics summarise the data.

**Result(s):**

200 US pts (62% male, mean age 40.4 years) and 556 EUR pts (57% male, mean age 38.9 years) completed the survey, with 77% and 54% currently receiving advanced therapies (biologic or novel oral therapy), respectively. The top 3 symptoms currently (past month) experienced by US and EUR pts were diarrhoea (63% and 50%), bowel urgency (47% and 30%) and increased stool frequency (39% and 30%). In past 3 months, pts who have ever experienced bowel urgency or urge incontinence reported bowel urgency (93% US, 89% EUR) and urge incontinence (86% US, 71% EUR) at least once a month (Table). 69% and 65% of all US and EUR pts, respectively, reported wearing a diaper/pad/protection at least once a month in the past 3 months due to fear/anticipation of urge incontinence. For pts receiving advanced therapies, similar patterns were observed. Among both US and EUR pts, the most common UC-related reasons for declining participation in social events were bowel urgency (43% and 30%) and fear of urge incontinence (40% and 32%). Similarly, the most common reasons for declining participation in work/school and sports/physical exercise were bowel urgency and fear of urge incontinence.

**Image:**

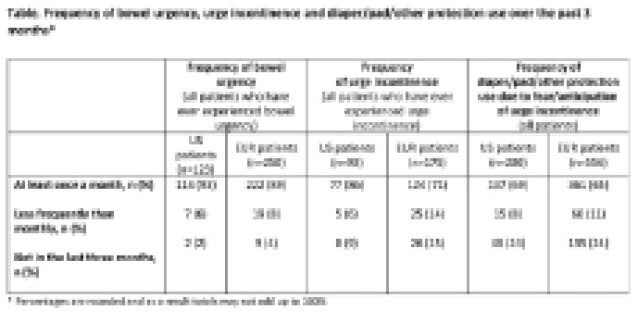

**Conclusion(s):**

Bowel urgency, which was the second-most frequently reported symptom, has an extensive impact on the lives of pts with moderate to severe UC. In this younger pt population, including pts receiving advanced therapies, almost two thirds of US and EUR pts reported wearing diapers/pads/protection at least once a month in the past 3 months due to fear/anticipation of urge incontinence. Both US and EUR pts reported bowel urgency and fear of urge incontinence as the top reasons for declining participation in social events, work/school, and sports/physical exercise.

**Please acknowledge all funding agencies by checking the applicable boxes below:**

Other

**Please indicate your source of funding;:**

Eli Lilly and Company

**Disclosure of Interest:**

S. Schreiber Grant / Research support from: personal fees and/or travel support from: AbbVie, Amgen, Arena Pharmaceuticals, Biogen, Bristol Myers Squibb, Celgene, Celltrion, Eli Lilly and Company, Dr. Falk Pharma, Ferring Pharmaceuticals, Fresenius Kabi, Galapagos NV, Gilead Sciences, I-MAB Biopharma, Janssen, Merck Sharp & Dohme, Mylan, Novartis, Pfizer, Protagonist Therapeutics, Provention Bio, Roche, Sandoz/Hexal, Shire, Takeda, Theravance Biopharma, and UCB Pharma, A. Bleakman Employee of: Eli Lilly and Company, M. Dubinsky Shareholder of: Trellus Health, Grant / Research support from: AbbVie, Janssen, Pfizer, and Prometheus Biosciences, Consultant of: AbbVie, Arena Pharmaceuticals, Boehringer Ingelheim, Bristol Myers Squibb, Celgene, Eli Lilly and Company, F. Hoffmann-La Roche, Genentech, Gilead Sciences, Janssen, Pfizer, Prometheus Therapeutics and Diagnostics, Takeda, and UCB Pharma, D. Rubin Grant / Research support from: Takeda, Consultant of: AbbVie, Allergan, AltruBio, American College of Gastroenterology, Arena Pharmaceuticals, Athos Therapeutics, Bellatrix Pharmaceuticals, Boehringer Ingelheim, Bristol Myers Squibb, Celgene/Syneos Health, Cornerstones Health (non-profit), Eli Lilly and Company, Galen/Atlantica, Genentech/Roche, Gilead Sciences, GoDuRn, InDex Pharmaceuticals, Ironwood Pharmaceuticals, Iterative Scopes, Janssen, Materia Prima, Pfizer, Prometheus Therapeutics and Diagnostics, Reistone Biopharma, Takeda, and TechLab, T. Hibi Grant / Research support from: AbbVie, Activaid, Alfresa Pharma, Bristol Myers Squibb, Eli Lilly Japan K.K., Ferring Pharmaceuticals, Gilead Sciences, Janssen Pharmaceutical K.K., JMDC, Nippon Kayaku, Mochida Pharmaceutical, Pfizer Japan, and Takeda, Consultant of: AbbVie, Apo Plus Station, Bristol Myers Squibb, Celltrion, EA Pharma, Eli Lilly and Company, Gilead Sciences, Janssen, Kyorin, Mitsubishi Tanabe Pharma, Nichi-Iko Pharmaceutical, Pfizer, Takeda, and Zeria Pharmaceutical, Speakers bureau of: AbbVie, Aspen Japan K.K., Ferring Pharmaceuticals, Gilead Sciences, Janssen, JIMRO, Mitsubishi Tanabe Pharma, Mochida Pharmaceutical, Pfizer, and Takeda, R. Panaccione Grant / Research support from: AbbVie, Ferring Pharmaceuticals, Janssen, Pfizer, and Takeda, Consultant of: Abbott, AbbVie, Alimentiv, Amgen, Arena Pharmaceuticals, AstraZeneca, Biogen, Boehringer Ingelheim, Bristol Myers Squibb, Celgene, Celltrion, Cosmo Pharmaceuticals, Eisai, Elan Pharma, Eli Lilly and Company, Ferring Pharmaceuticals, Galapagos NV, Genentech, Gilead Sciences, GlaxoSmithKline, Janssen, Merck, Mylan, Oppilan Pharma, Pandion Therapeutics, Pfizer, Progenity, Protagonist Therapeutics, Roche, Sandoz, Satisfai Health, Shire, Sublimity Therapeutics, Takeda, Theravance Biopharma, and UCB Pharma, T. Gibble Employee of: Eli Lilly and Company, C. Kayhan Employee of: Eli Lilly and Company, E. Flynn Employee of: Eli Lilly and Company, C. Sapin Employee of: Eli Lilly and Company, C. Atkinson Consultant of: Eli Lilly and Company in connection with the development of this publication, Employee of: Adelphi Real World, S. Travis Grant / Research support from: AbbVie, BUHLMANN Diagnostics, ECCO, Eli Lilly and Company, Ferring Pharmaceuticals, International Organization for the Study of Inflammatory Bowel Disease, Janssen, Merck Sharp & Dohme, Normal Collision Foundation, Pfizer, Procter & Gamble, Schering-Plough, Takeda, UCB Pharma, Vifor Pharma, and Warner Chilcott, J. Jones: None Declared

